# Sulfur-Fumigated Ginger Identification Method Based on Meta-Learning for Different Devices

**DOI:** 10.3390/foods13233870

**Published:** 2024-11-29

**Authors:** Tianshu Wang, Jiawang He, Hui Yan, Kongfa Hu, Xichen Yang, Xia Zhang, Jinao Duan

**Affiliations:** 1 College of Artificial Intelligence and Information Technology, Nanjing University of Chinese Medicine, Nanjing 210023, China; daaang@126.com (J.H.); kfhu@njucm.edu.cn (K.H.); 2 Jiangsu Province Engineering Research Center of TCM Intelligence Health Service, Nanjing University of Chinese Medicine, Nanjing 210023, China; 3 National and Local Collaborative Engineering Center of Chinese Medicinal Resources Industrialization and Formulae Innovative Medicine, Nanjing University of Chinese Medicine, Nanjing 210023, China; zhangxia@njucm.edu.cn (X.Z.); dja@njucm.edu.cn (J.D.); 4 College of Computer Science and Technology, Nanjing Normal University, Nanjing 210023, China; xichen_yang@njnu.edu.cn

**Keywords:** ginger, sulfur fumigation, image processing, deep learning, meta-learning

## Abstract

Since ginger has characteristics of both food and medicine, it has a significant market demand worldwide. To effectively store ginger and achieve the drying and color enhancement effects required for better sales, it is often subjected to sulfur fumigation. Although sulfur fumigation methods can effectively prevent ginger from becoming moldy, they cause residual sulfur dioxide, harming human health. Traditional sulfur detection methods face disadvantages such as complex operation, high time consumption, and easy consumption. This paper presents a sulfur-fumigated ginger detection method based on natural image recognition. By directly using images from mobile phones, the proposed method achieves non-destructive testing and effectively reduces operational complexity. First, four mobile phones of different brands are used to collect images of sulfur- and non-sulfur-fumigated ginger samples. Then, the images are preprocessed to remove the blank background in the image and a deep neural network is designed to extract features from ginger images. Next, the recognition model is generated based on the features. Finally, meta-learning parameters are introduced to enable the model to learn and adapt to new tasks, thereby improving the adaptability of the model. Thus, the proposed method can adapt to different devices in its real application. The experimental results indicate that the recall rate, F1 score, and AUC-ROC of the four different mobile phones are more than 0.9, and the discrimination accuracy of these phones is above 0.95. Therefore, this method has good predictive ability and excellent practical value for identifying sulfur-fumigated ginger.

## 1. Introduction

The market demand for ginger is considerable worldwide. Large populations, especially in Asia, Africa, and Europe, have the habit of eating ginger daily. Ginger (*Zingiberis rhizoma*) is used in a variety of foods, such as ginger soup, gingerbread, and ginger drinks [1,2]. It is also a common medicine for health care [3,4]. However, polysaccharides, vitamins, and water inside ginger are suitable for microbial reproduction and are prone to mold. To prevent ginger from becoming moldy and maintain its bright color, it is common to fumigate ginger with sulfur. This process can easily lead to excessive sulfur dioxide, which is seriously harmful to the human body [5,6]. The residual sulfur dioxide in herbal medicine can not only cause bad taste, but can also cause respiratory symptoms such as coughing, chest tightness, and throat irritation [7]. At the same time, residual sulfur dioxide within ginger may react chemically with other ingredients, changing the composition or content of the effective ingredients, thereby affecting the quality of ginger [8]. Therefore, it is necessary to conduct quality testing on ginger to determine whether it is sulfur-fumigated.

Currently, the identification methods of the sulfur fumigation process mainly include HPLC (high-performance liquid chromatography) fingerprint methods [9,10], infrared spectroscopy analysis [8], and gas chromatography methods [11]. At present, a large number of research results have been accumulated by using HPLC to study the composition changes of food and medicines before and after sulfur fumigation. Ma et al. analyzed sulfur-fumigated and air-dried Codonopsis Radix samples and found significant differences in chemical composition [12]. Wang et al. analyzed non-fumigated and sulfur-fumigated chrysanthemums. The results show that the contents of four flavonoid aglycones significantly increase and those of seven glycosides significantly decrease [13]. These studies show that the HPLC methods can effectively distinguish sulfur- and non-sulfur-fumigated materials. Infrared spectroscopic analysis has the advantage of being fast and non-destructive and is also widely used to identify sulfur-fumigated foods. He et al. evaluated the degree of sulfur fumigation by detecting the residual sulfur dioxide in Fritillaria thunbergii Bulbus [14]. Yan et al. established a method to distinguish sulfur-fumigated ginger from ginger samples based on Fourier transform near-infrared spectroscopy [15]. These studies demonstrate that sulfur fumigation can be quickly identified through infrared spectroscopy, providing an idea for the identification of sulfur-fumigated foods. The academic community also collects gas chromatograms of food or medicinal materials to identify sulfur-smoked products. Zhang et al. used Heracles NEO ultra-fast gas phase electronic nose technology to analyze raw and sulfur-fumigated Paeoniae Radix Alba decoction pieces in order to establish a rapid identification method for sulfur-fumigated Paeoniae Radix Alba [16]. However, these methods require professional training and require highly sophisticated equipment to extract and analyze the chemical components of a sample. Therefore, only experienced technicians with professional skills and instrument operation experience are suitable for these tasks. Due to their high professional requirements, not all laboratories or production environments can easily apply these technologies. Although it has performed well in the quality control of sulfur fumigation products, there are still challenges in popularity. These traditional sulfur detection methods (HPLC, infrared spectroscopy, and gas chromatography) face disadvantages such as complex operation, high time consumption, and easy consumption.

In recent years, artificial intelligence (AI) has become a research hotspot in the development of science and technology [17,18]. With the development of information technology such as multimedia, the application of smartphones has become highly popular. The popularity of smartphones provides a prerequisite for taking images of ginger to conduct intelligent analysis of sulfur fumigation with the help of visual information. In the fields of the identification and quality inspection of food or medicine, artificial intelligence technologies, such as image processing and deep learning, have received great attention [19,20,21,22]. Zhang et al. used an algorithm that combines deep learning with machine vision to automatically identify the origin of 1859 Angelica sinensis samples, which were obtained from eight regions [23]. Liu et al. applied the deep learning models VGG16 and ResNet50 to identify chrysanthemum cultivar [24]. Haq et al. used the Convolution Neural Network to detect weeds in crops [25]. Haq et al. used the Random Forest algorithm to identify vegetation types [26]. It can be inferred that deep learning technology can help extract useful features from a large number of ginger images and use these features for classification and identification. Therefore, the method based on image processing technology combined with deep learning has broad prospects for identifying sulfur-fumigated ginger. More importantly, compared to traditional sulfur detection methods, AI technology achieves non-destructive testing while effectively reducing operational complexity, saving detection time and resource consumption.

In reality, however, the images taken by different mobile phones have large differences in chroma, saturation, resolution, etc. The established recognition models based on images that are collected from a single device are not suitable for all application scenarios. Therefore, it is necessary to develop a sulfur-fumigated ginger identification model based on images from multiple devices. In this case, meta-learning algorithms can play an important role. Meta-learning is technology that learns to learn. This technology can rapidly adapt to new tasks [27,28,29]. To diagnose bearing faults in rotating machinery, Cheng et al. used a model-agnostic meta-learning method to learn the parameters of a neural network in a source domain dataset to quickly adapt to the target domain dataset [30]. Pang et al. proposed an industrial product surface defect detection algorithm based on meta-learning and neural networks without extensive pre-training and additional image input [31]. Therefore, it is feasible to introduce meta-learning to improve the identification model. Therefore, given the strong learning ability of meta-learning for multitasking and small sample datasets, this architecture will effectively achieve the recognition of different mobile phone ginger images, avoid image specificity issues, and improve the universality of the model. Thus, this meta-learning framework achieves the effective identification of images captured by different mobile phones.

For the identification of sulfur-fumigated ginger, meta-learning can help the model better adapt to images taken by different mobile phones. By training images from multiple different devices, meta-learning can enable the model to better learn the differences between different devices and can process new data through the learned patterns. In this way, the established model can better adapt to new scenarios and further improve the accuracy of the model. Therefore, this paper presents a sulfur-fumigated ginger identification method based on meta-learning. The established model adapts to images from different devices and is more suitable for practical application scenarios. The main contributions of this study are as follows:

A ginger image database is set up. The database includes ginger images taken by four different mobile phones: iPhone, Xiaomi, Honor, and vivo. All the images are labeled with sulfur fumigation or non-sulfur fumigation. Collecting images through these four representative mobile phones can help achieve a more extensive and objective recognition of sulfur-fumigated ginger.

An effective feature analysis method for fumigated ginger is presented. The Faster R-CNN model is introduced to effectively remove the background noise of the images. A hybrid multi-layer deep network is designed to learn and extract the image features of sulfur- and non-sulfur-fumigated ginger samples.

Meta-learning technology is adopted to establish a sulfur-fumigated ginger identification model, which is suitable for images from different devices, thereby improving the generalization ability of the model. This method ensures high accuracy and stability in identifying sulfur-fumigated ginger.

The model performs well in identifying ginger images from different cellphones, with accuracy rates above 90%. The model has significantly improved performance and accuracy compared with other methods. This work is expected to bring wider application and development to the identification of sulfur-fumigated foods or medicinal materials.

## 2. Materials and Methods

**Figure 1:**
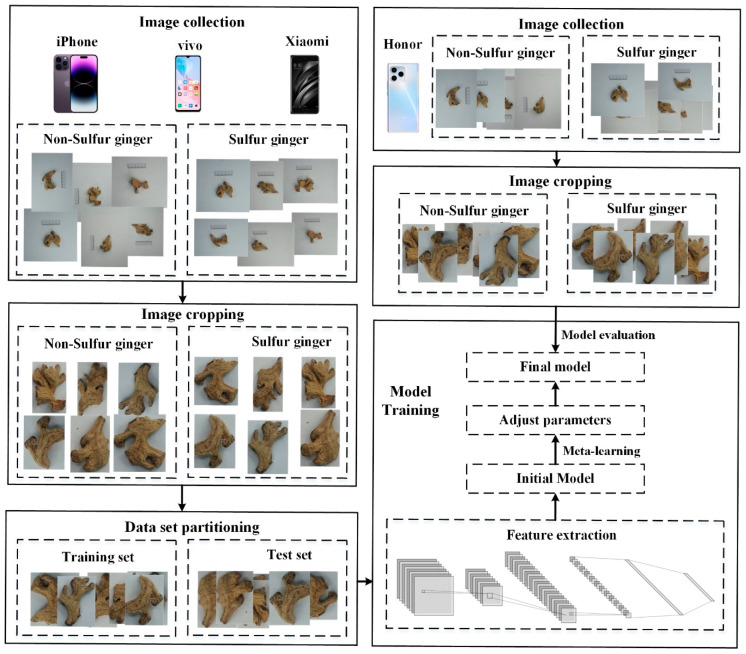
Flow chart of the proposed method.

In this paper, a sulfur-fumigated ginger identification method based on meta-learning for different devices is proposed. The flow chart is illustrated in Figure 1. First, a large number of sulfur- and non-sulfur-fumigated ginger images were collected through different devices, and a ginger image database with sulfur-fumigation information was set up. Next, a deep learning model was used to train an intelligent identification model that can accurately identify sulfur-fumigated ginger. To improve the applicability of the model for different devices, meta-learning was then introduced to optimize the model parameters. Finally, the model was analyzed and evaluated through multiple metrics.

### 2.1. Sample Preparation and Image Acquisition

Sulfur- and non-sulfur-fumigated ginger samples were purchased from the market. The images of the ginger samples were collected using an iPhone 14 Pro Max (Apple Inc., Cupertino, CA, USA), vivo Y76s (Vivo Mobile Communication Co., Ltd., Dongguan, China), Honor 60 SE (Honor Device Co., Ltd., Shenzhen, China), and Xiaomi 6 (Xiaomi Technology Co., Ltd., Beijing, China). The camera parameters of these phones are shown in Table 1. These phones represent different levels of the current mobile phone market. The iPhone 14 Pro Max represents high-end models with top shooting performance and technology. The Honor 60 SE and vivo Y76s represent mid-range models with the Android system, and these types of phone models have the highest market share. In addition, many old models still have a group of loyal users, so the addition of the Xiaomi 6 can better adapt to this user group.

The image information of ginger simples is illustrated in Table 2. There were 536 images in the database, of which there were 264 images of sulfur-fumigated ginger samples, with each phone model representing 66 images, and there were 264 images of non-sulfur-fumigated ginger samples, with each phone model representing 68 images. In Table 2, iPh represents iPhone, Vo represents vivo, Ho represents Honor, Mi represents Xiaomi, NS represents non-sulfur-fumigated ginger, and HS represents sulfur-fumigated ginger.

**Figure 2:**
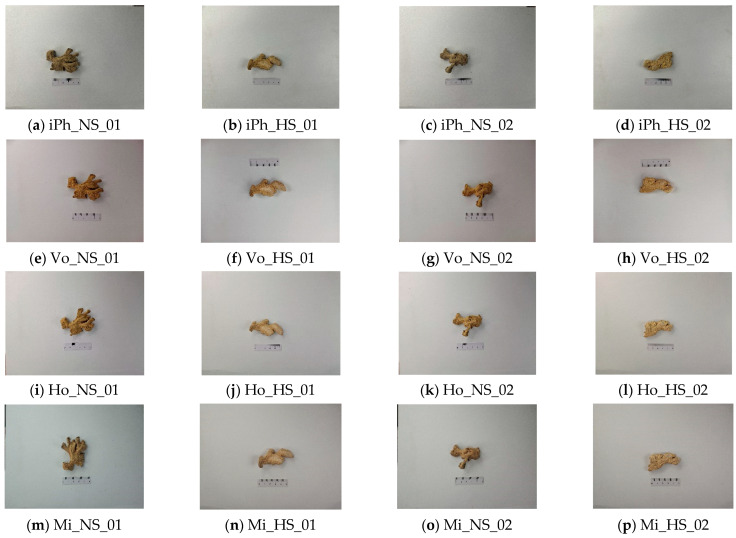
Image of ginger samples shot by four types of mobile phones.

The images of ginger samples shot by the four mobile phones are shown in Figure 2. It can be seen that it is difficult to identify sulfur- and non-sulfur-fumigated ginger samples with the naked eye. In addition, there are certain differences between the images from different mobile phones for the same ginger sample.

### 2.2. Image Cropping

As shown in Figure 2, the proportion of ginger in the images is relatively small, and there is a large amount of blank background space. The blank area will affect the training and recognition of the model. Therefore, it is necessary to preprocess the images to remove invalid background information. In this study, the Faster R-CNN model [32] with a ResNet-50-FPN backbone is used for ginger detection. This model combines multiple network structures and technologies to achieve efficient and accurate target detection.

**Figure 3:**
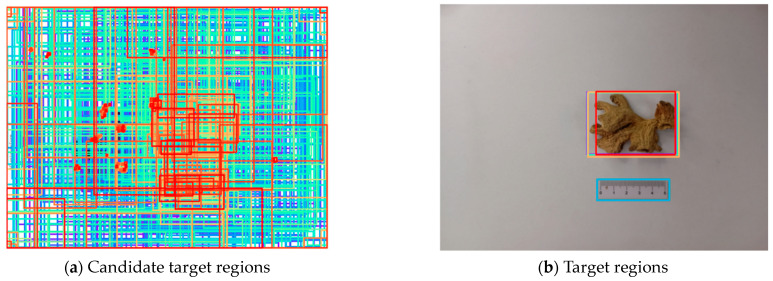
Example of candidate target regions.

Firstly, the size of original ginger images is uniform since the images from different devices have different resolutions. The resized image is input into the ResNet50 network to generate feature maps of different levels. FPNs (Feature Pyramid Networks) can integrate feature maps of different levels to enhance semantic information. Then, these feature maps are input into FPNs to generate new multi-scale feature maps. The new feature maps obtained can be used to detect large-, medium-, and small-scale targets at the same time. Next, the region proposal networks are adopted to explore potential candidate target regions based on the feature maps. An example of the candidate target regions is shown in Figure 3a, where the colorful boxes are the candidate target regions. There are 1000 candidate target regions of different scales in Figure 3a.

After that, the region-of-interest pooling process is performed to obtain 1000 feature vectors with a uniform size, where each feature vector represents its corresponding candidate target region. Then, the categories and coordinates of the regions are calculated through softmax and regression. Next, the suitable target regions are screened out by deleting the invalid regions, which are regarded as background regions or tiny-scale regions. Figure 3b illustrates the four selected target regions. Three boxes represent the ginger region, and the other one is the ruler region.

**Figure 4:**
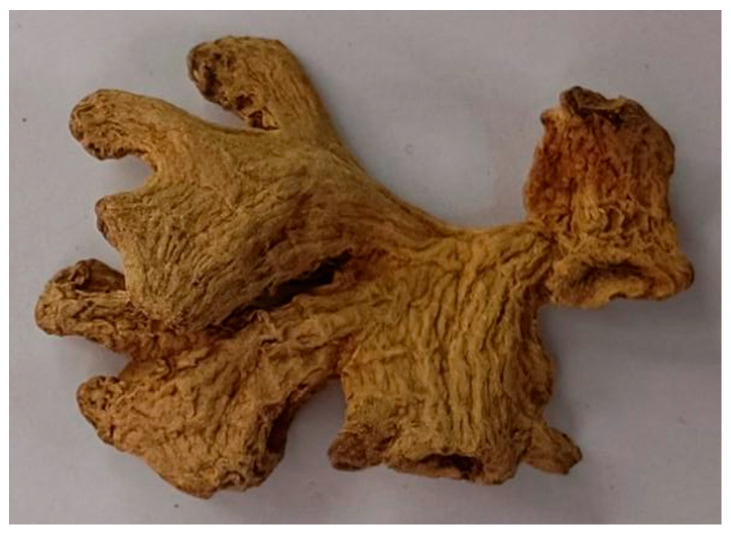
Final cropping result.

As shown in Figure 3b, the ginger boxes are larger than the ruler box, and the largest among the ginger boxes covers the most complete ginger information. In this study, the largest region is selected. Finally, the ginger region can be cropped out from the original ginger image based on the coordinates of the region boxes. Figure 4 shows the final cropped ginger region. As shown in Figure 4, the background is successfully removed, and the ginger region remains intact.

### 2.3. Initial Model

This study uses ginger images taken by iPhone, vivo, and Xiaomi phones to train the sulfur-fumigated ginger identification model. Let S be the sample space, train be the training set samples, and test be the test set samples. train and test are related as shown in Formulas (1) and (2), where N(train) represents the number of training set samples and N(test) represents the number of test set samples.

(1) train∩test=∅

(2) train∪test=S

**Figure 5:**
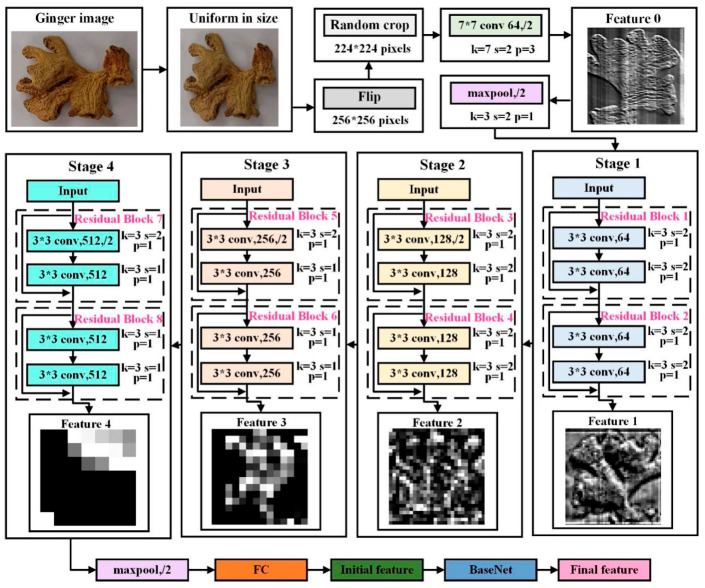
The flow chart of feature extraction.

Sulfur- and non-sulfur-fumigated ginger images have certain differences in color and texture, which can be captured and expressed by deep learning networks. Thus, the features are extracted through deep neural networks, as shown in Figure 5. To unify the image size and reduce computational complexity, the images in the training set need to be transformed. First, the image size is uniformly adjusted to 256 × 256 pixels. Then, the image is horizontally flipped with a probability of 50% to increase the generalization ability of the model. To introduce some randomness and prevent the model from overfitting, a random area of 224 × 224 pixels is cropped from the image. Next, the image is normalized using Formula (3) to make the model training more stable.

(3) $$normalized\_image=\frac{image-\mu }{\sigma }$$

where μ represents the mean of the image and σ represents the standard deviation of the image. μ and σ are calculated by Formulas (4) and (5), respectively.

(4) $$\mu =\frac{\sum_{i=1}^{w}\sum_{j=1}^{h}\left(p_{ij}\right)}{w*h}$$

(5) $$\sigma =\sqrt{\frac{\sum_{i=1}^{w}\sum_{j=1}^{h}(p_{ij}-\mu {)}^{2}}{w*h}}$$

where w denotes the width of the image, h represents the height of the image, and $p_{ij}$ denotes the pixel value of the ij position in the image.

After that, a pre-trained deep convolutional neural network ResNet18 [33] is applied to extract the image features. First, the image is processed by a convolution operation with kernel size *k*, stride *s*, and padding *p* set to 7, 2, and 3, respectively. Then, the average pooling operation is performed with *k* = 3, *s* = 2, and *p* = 1. Next, there are four stages to process the input information. Each stage contains two residual blocks, each of which contains two convolutional layers, using jump connections to transfer features. These connections not only help adjust the dimension of the feature map but also maintain the integrity of the features, thereby improving the learning ability of the network without adding additional computational burden. Finally, the final average pooling layer and the fully connected layer generate the final feature.

The feature maps, Features 0 to 4 in Figure 5, reflect how the key information of the image at different levels is captured and expressed. It can be observed that, as the network level deepens, the shapes and structures in Features 0 to 4 become more abstract, and Feature 4, which is generated after Stage 4, summarizes and simplifies the information of the image.

Next, a typical fully connected neural network named BaseNet is designed to further learn the initial feature. BaseNet contains three fully connected layers (FC1, FC2, FC3), two Batch Normalization layers (BN1, BN2), two Dropout layers (Drop1, Drop2), a PReLU activation function, and a Sigmoid activation function. Among them, Dropout layers are used to limit the complexity of the model, improve its generalization ability, and thus avoid overfitting. These components work together to transform the initial feature to the final feature, and then determine whether the ginger is fumigated. The structure of BaseNet is shown in Table 3.

Combining the advantages of ResNet18 and BaseNet, a hybrid model is created. The deep convolutional layers of ResNet18 can extract rich features from the input data, while the fully connected layers of BaseNet can use these features for effective classification. In addition, the Dropout and BN layers in BaseNet can prevent overfitting, thereby improving the generalization ability of the model. Next, the mean square error loss is set as the loss function and Adam is set as the optimizer. This optimizer contains the parameters of two models: ResNet18 and BaseNet.

### 2.4. Final Model Based on Meta-Learning

In this study, meta-learning parameters are introduced to improve the recognition accuracy of the model since the processed data are derived from various mobile phones. First, the images from the iPhone, vivo, and Xiaomi mobile phones are taken as the support and query sets, which are not disjointed. ${Sup}^{i}$ and ${Que}^{i}$, respectively, denote the support and query sets for the *i*-th mobile phone. Then, the meta-training dataset ${Meta}^{i}$ can be obtained through Formula (6).

(6) $${{Meta}^{i}=\{{Sup}^{i},{Que}^{i}\}}_{i=1}^{n}$$

where *n* represents the number of mobile phones which participate in the training process. In this study, *n* is equal to 3. The model trained by ${Sup}^{i}$ is denoted as $M_{Sup}^{i}$ and the model trained by ${Que}^{i}$ is denoted as $M_{Que}^{i}$.

To minimize the difference between the prediction value and the real value, the loss function ζ is calculated as follows:

(7) $$\zeta =\left\|f\right.\left(x\right)-{\left.y\right\|}_{2}^{2}$$

In this study, if the ginger in the image is sulfur-fumigated, the real value of the image is set as 0. Otherwise, the value is set as 1. $f\left(x\right)$ denotes the predicted value by the model for the input image *x*. y denotes the real value of the image *x*.

Next, the model parameters are updated by

(8) $$\vartheta_{Sup}^{i}=\vartheta_{Que}^{i-1}-\partial_{1}\sum_{o=1}^{O}\frac{m_{Sup}^{i,o}}{\varepsilon +\sqrt{v_{Sup}^{i\_o}}}$$

(9) $$\vartheta_{Que}^{i}=\vartheta_{Sup}^{i}-\partial_{1}\sum_{o=1}^{O}\frac{m_{Que}^{i,o}}{\varepsilon +\sqrt{v_{Que}^{i,o}}}$$

where $\vartheta_{Sup}^{i}$ and $\vartheta_{Que}^{i}$ are the model parameters of $M_{Sup}^{i}$ and $M_{Que}^{i}$, respectively. $\vartheta_{Sup}^{1}$ is the parameter of the initial model. $\partial_{1}$ denotes the internal learning rate. ε is a constant, $1*10^{-8}$. O is the number of gradient updates. $m_{Sup}^{i,o}$ and $m_{Sup}^{i,o}$ are the first-order moments of the gradient at the *o*-th step, while $v_{Sup}^{i,o}$ and $v_{Sup}^{i,o}$ are the second-order moments of the same:

(10) $$m_{Sup}^{i,o}=\varphi_{1}m_{Sup}^{i,o-1}+({1-\varphi }_{1}){▽}_{\vartheta }\zeta$$

(11) $$m_{Que}^{i,o}=\varphi_{1}m_{Que}^{i,o-1}+({1-\varphi }_{1}){▽}_{\vartheta }\zeta$$

(12) $$v_{Sup}^{i,o}=\varphi_{2}v_{Sup}^{i,o-1}+({1-\varphi }_{2}){▽}_{\vartheta }\zeta^{2}$$

(13) $$v_{Que}^{i,o}=\varphi_{2}v_{Que}^{i,o-1}+({1-\varphi }_{2}){▽}_{\vartheta }\zeta^{2}$$

where $\varphi_{1}$ and $\varphi_{2}$ denote the exponential decay rates.

Let M′ denote the model after learning iPhone, vivo, and Xiaomi mobile phones and ϑ′ denote parameters of M′. Finally, $M^{F}$ are fine-tuned by the images from the Honor phone, and the parameters of $M^{F}$ are denoted as $\vartheta^{F}$, which is calculated as

(14) $$\vartheta^{F}=\vartheta \prime -\partial_{F}\sum_{t=1}^{T}\frac{m^{t}}{\varepsilon +\sqrt{v^{t}}}$$

where $\partial_{F}$ denotes the internal learning rate during fine-tuning. T is the number of gradient updates for fine-tuning. $m^{t}$ and $v^{t}$ are the first- and second-order moments of the gradient at the *t*-th step, respectively.

## 3. Results

The hardware environment of the experiment is a computer (Xiaoxin Pro13 2020 Ryzen version, Lenovo, Beijing, China) equipped with an AMD Ryzen 5 4600U CPU, 16 GB of DDR4 3200 MHz memory, and the Windows 10 operating system. The programming language is Python. The experimental data include 536 ginger images, which were taken by four different mobile phones: the iPhone 14 Pro Max, vivo Y76s, Honor 60 SE, and Xiaomi 6. Each phone provides 134 sample pictures.

### 3.1. Evaluation Metrics

To comprehensively evaluate the performance of the prediction model, this study calculates a series of key evaluation metrics: precision, recall, F1 score, AUC-ROC (Area Under the Curve of the Receiver Operating Characteristic), and accuracy. The meanings of these parameters are shown in Table 4. These metrics evaluate the performance of the model from different perspectives. Precision, recall, and accuracy evaluate the classification performance of the model from the perspectives of prediction accuracy and coverage. Through these metrics, the performance of the model can be fully learned.

### 3.2. Performance Evaluation

In the performance evaluation experiment, the ratio of the training set to the test set is 8:2. And the train–test procedure is repeated 200 times to guarantee the stability and robustness of the model. In each train–test procedure, there is no dataset overlap. The model evaluation metrics are illustrated in Table 5. As shown in Table 5, the recall, F1 score, and AUC-ROC of all mobile phones are over 0.9. This indicates that the model performs well in identifying actual positive samples, and the model achieves high precision values on iPhone, vivo, and Xiaomi mobile phones, with all being greater than 0.95. For the Honor mobile phone, the images do not participate in model training, but its precision value still reaches 0.83.

**Figure 6:**
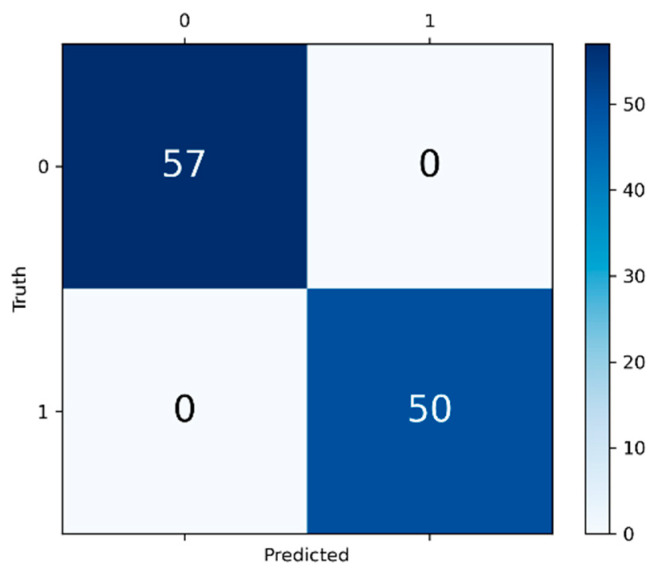
Confusion matrix.

The confusion matrix is illustrated in Figure 6. The final output is discrete label 0 or 1, where 0 represents non-sulfur-fumigated ginger, and 1 represents sulfur-fumigated ginger. Fifty-seven positive samples and fifty negative samples are correctly identified. This result indicates that the model has reached 100% accuracy on the dataset. This high accuracy reflects a strong identification ability. Combining the evaluation metrics and the confusion matrix, it is demonstrated that the model proposed in this paper has high generalization ability and can adapt to different mobile phones.

### 3.3. Proportional Sensitivity

**Figure 7:**
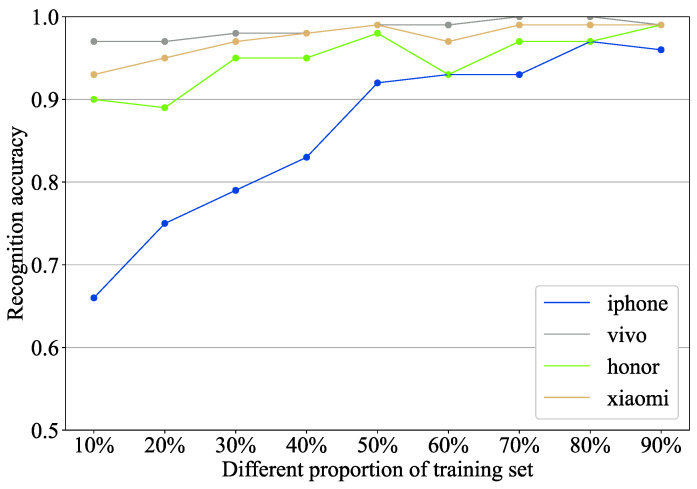
Average accuracy of different training set ratios.

The proportion of the training set is a key factor that directly affects the accuracy of the prediction results. To evaluate the sensitivity of the model to the proportion of the training set, this study designs nine groups of comparative experiments. The proportion of the training set is changed to 0.1, 0.2, 0.3, 0.4, 0.5, 0.6, 0.7, 0.8, and 0.9. The train–test procedure for each ratio is repeated 200 times to ensure the fairness of the results. Figure 7 shows the average accuracy of the model under different training set ratios.

For the iPhone, the accuracy ranges from 0.69 to 0.95 at training set ratios of 0.1 to 0.9. As the training set ratio increases, the accuracy shows an upward trend. This is because, as the proportion of the training set increases, the model learns more samples, thereby increasing its ability to distinguish sulfur-fumigated ginger. For vivo and Xiaomi phones, the accuracy is about 0.97 to 0.99 and 0.9 to 0.99, respectively. It is worth noting that the model of Honor is generated by fine-tuning the model of three other mobile phones through the meta-learning framework. In Figure 7, the average accuracy of Honor can still reach more than 0.9. This result indicates that the method proposed in this paper is effective and has good practicality. It is feasible to identify the sulfur-fumigated ginger for new mobile phones through the meta-learning framework.

### 3.4. Stability Analysis

**Figure 8:**
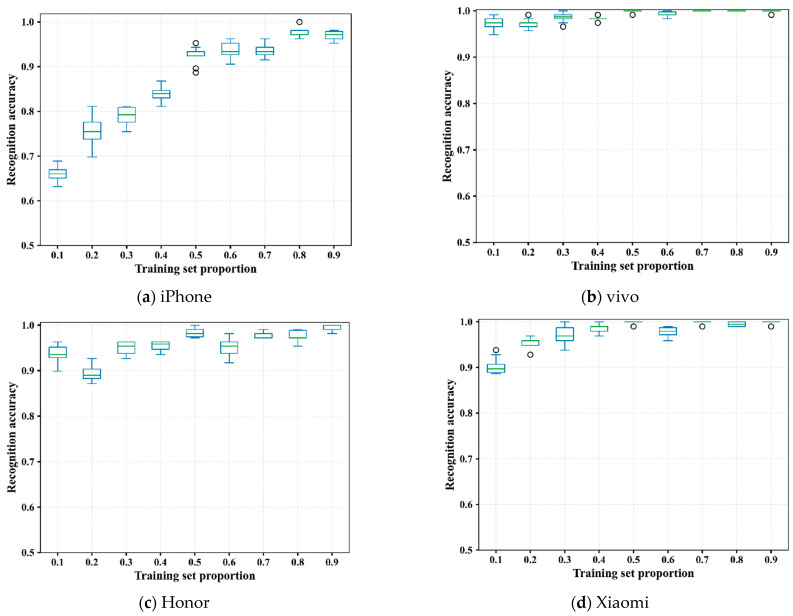
Boxplots of accuracy distributions within 200 iterations of four mobile phones at different training set proportions.

Stability is also a key factor in evaluating the model. Figure 8 illustrates the boxplots of accuracy distributions of iPhone, vivo, Honor, and Xiaomi mobile phones at different training set ratios. For each box, the central green line is the median value of the 200 train–test procedures. It can be seen that the fluctuation range of accuracy within 200 iterations for all the mobile phones is small. For the vivo phone, the fluctuation in accuracy is smallest at all training set ratios, almost approaching or reaching 1. Therefore, the model proposed in this paper has good stability.

### 3.5. Performance Comparison

During the training process of the model, different learning rates have an impact on the convergence and training effectiveness of the model. By setting different learning rates, we obtained the discrimination results of different models. Table 6 illustrates the accuracy comparison results of models with different learning rates in the proposed method. From Table 6, it can be seen that when the learning rate of the model is set to 0.01, the prediction accuracy is highest. When the learning rate is set to 0.1 and 1, the performance of the models is not satisfactory. Thus, 0.01 is selected as the learning rate of the proposed method.

Similarly, different model optimizers play a crucial role in model performance. Table 7 illustrates the accuracy comparison results of models with different optimizers in the proposed method. From Table 7, it can be seen that the optimizer Adaptive Moment Estimation (Adam) performs better than the other three optimizers, namely Stochastic Gradient (SGD), Average Stochastic Gradient Descent (ASGD), and Adaptive Gradient (Adagrad). Therefore, Adam is employed in the proposed method.

In order to better evaluate the performance of the model, this study tests the model on two experiment environments with different configurations. Table 8 illustrates the running time comparison of models with different experimental environments in the proposed method. As shown in Table 8, the CPU of experiment environment 1 is AMD Ryzen 5 4600U, the training time of the model is 125 s, and the prediction time of the test set is 20 s. The CPU of experiment environment 2 is AMD Ryzen 7 3750H. The training time of the model is 194 s, and the prediction time of the test set is 23 s. The running time of the two models is close and the time consumption is less, which shows that the model has good generalization and detection speed.

**Figure 9:**
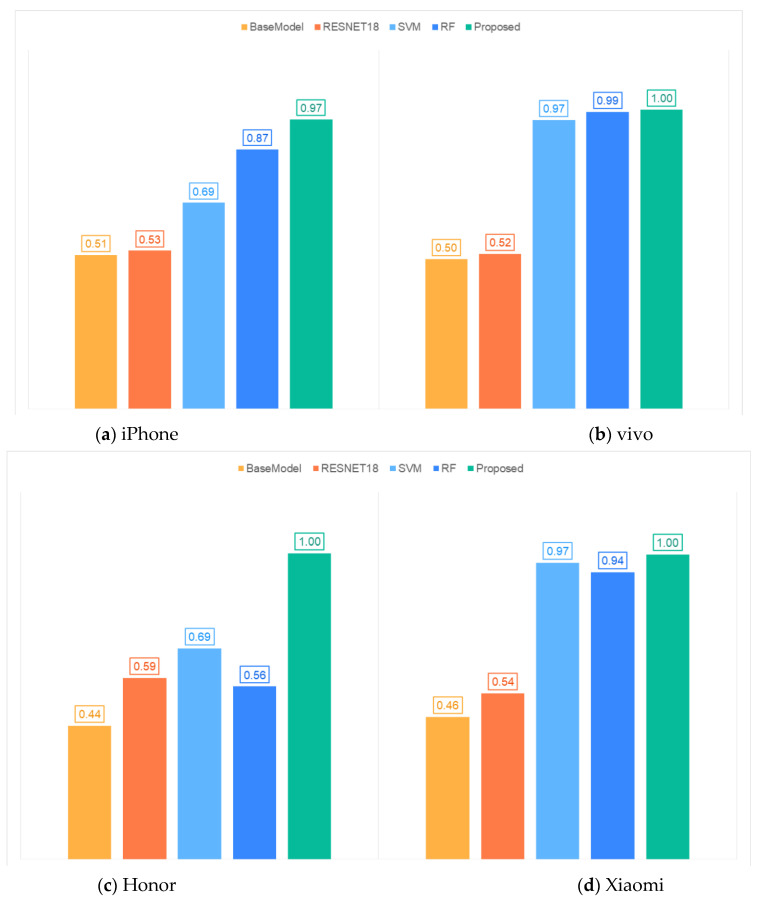
Accuracy of five different methods on four mobile phone models.

In addition, the proposed method is compared with four methods. These methods are BaseModel, ResNet18, SVM (Support Vector Machine) [34], and RF (Random Forest) [35]. In the BaseModel and ResNet18 methods, BaseNet and ResNet18 are used for feature extraction. In the SVM and RF methods, the Hu-invariant moments [36,37] and Gabor transform features [38,39] are used for feature extraction. The parameter settings for SVM and RF are the same as the settings in the reference [40]. The loss function of the SVM model is set to 100, and the gamma function in the kernel function is set to 0.1. In the RF model, the nTree parameter is set to 2000 and the entry parameter is set to 50. The ratio of the training set to test set is 8:2, and the train–test procedure of each method is repeated 200 times. Figure 9 illustrates the average accuracy of the five methods.

As shown in Figure 9, the accuracy of the BaseModel and ResNet18 methods on the four mobile phones did not exceed 0.6. This is probably because these two models cannot fully capture the diversity and complexity of the data when processing complex image datasets. The accuracies of the SVM and RF methods on the Honor mobile phone that did not participate in training were also less than 0.7. This is probably because the model failed to learn enough generalization features during the training process. The method proposed in this paper achieved the highest accuracy for all the mobile phones used. This proves that the model has significant capabilities in processing the images from different devices.

## 4. Discussion

In recent years, sulfur fumigation has been widely used in various traditional Chinese medicines as a low-cost, fast, and effective drying and color enhancing method. The residual sulfur dioxide in Chinese herbal medicines treated by this method inflicts a certain degree of harm to people’s health, and in severe cases it can damage the liver, kidneys, and other organs [41]. Therefore, it is necessary to identify sulfur fumigated herbs.

At present, many researchers have studied the identification methods of sulfur fumigated herbs [42,43]. Most of these studies use HPLC to detect the residual amount of sulfur dioxide in traditional Chinese medicine [44]. HPLC-based methods have the disadvantages of complex operation, high time consumption, and easy consumption. Yan et al. used near-infrared spectroscopy technology to quickly and non-destructively distinguish sulfur- and non-sulfur-fumigated ginger [15]. Compared with conventional HPLC, this method has the advantages of being rapid and non-destructive, but it requires professional instruments and personnel to operate.

The method proposed in this paper collects the images of dried ginger by mobile phone, extracts the image features, analyzes the differences between different types of images, and constructs the recognition model of sulfur-fumigation ginger. This image identification method does not need professional instruments and technicians, and also has the advantages of easy operation, speed, and is nondestructive. In addition, we use meta-learning to improve the adaptability of the model, so that the model can quickly adapt to the image information of different mobile phones, and effectively improve the stability and generalization ability of the model. The recognition accuracy of the method proposed in this paper is 0.97, 1, 1, and 1 for the sulfur-fumigated ginger photographed by the iPhone, vivo, or Xiaomi, respectively. The recognition performance is competitive compared with existing methods. Therefore, this method can effectively identify sulfur-fumigation ginger and has excellent practical value.

## 5. Conclusions

In the current market, ginger is in great demand. Traditional image-based fumigated-ginger identification methods are inapplicable to different devices. To solve this problem, this study has developed a sulfur-fumigated ginger identification method based on deep learning and meta-learning technology. The proposed method can adapt to different devices and can be widely used in actual application environments. The experimental results demonstrate that the proposed method has high accuracy on various mobile phones. The recall rates, F1 scores, and AUC-ROC values exceed 0.9. Therefore, the proposed method has excellent predictive performance and practicality.

Only four brands of mobile phones collected images of ginger in this study, which may give the model some specificity. In future research, we will collect more ginger image data from other phones, expand the data scale, and further train the model to achieve high stability, accuracy, and universality of the model.

## Figures and Tables

**Table 1:** Camera parameters of the mobile phones.

Phone Model	Megapixel	Aperture
iPhone 14 Pro Max	48	ƒ/1.78
vivo Y76s	50	ƒ/1.8
Honor 60 SE	50	ƒ/1.8
Xiaomi Mi 6	12	ƒ/1.8

**Table 2:** Image information of ginger samples.

Phone Model	Non-Sulfur Ginger	Sulfur Ginger	Resolution
iPhone 14 Pro Max	iPh_NS_01-iPh_NS_68	iPh_HS_01-iPh_HS_66	4032 × 3024 pixels
vivo Y76s	Vo_NS_01-Vo_NS_68	Vo_HS_0-Vo_HS_66	4080 × 3060 pixels
Honor 60 SE	Ho_NS_01-Ho_NS_68	Ho_HS_01-Ho_HS_66	4608 × 3056 pixels
Xiaomi Mi 6	Mi_NS_01-Mi_NS_68	Mi_HS_01-Mi_HS_66	4032 × 3016 pixels

**Table 3:** BaseNet structure.

Layer Name	Type	Input Size	Output Size
FC1	Linear Layer	1000	1024
BN1	Batch Normalization Layer	1024	1024
Drop1	Dropout Layer	1024	1024
FC2	Linear Layer	1024	512
BN2	Batch Normalization Layer	512	512
Drop2	Dropout Layer	512	512
FC3	Linear Layer	512	1

**Table 4:** Model evaluation metrics.

Index	Meaning
Precision	The proportion of samples predicted to be positive that are actually positive.
Recall	The proportion of samples that are actually positive that are predicted to be positive.
F1 Score	The harmonic mean of precision and recall.
AUC-ROC	Classification performance of the model at different thresholds.
Accuracy	The proportion of correct prediction results in the total samples.

AUC-ROC: Area Under the Curve of the Receiver Operating Characteristic.

**Table 5:** Model evaluation parameters.

Phone	Precision	Recall	F1 Score	AUC-ROC
iPhone	0.9672	0.9487	0.9518	0.9528
vivo	0.9944	1.0000	0.9972	0.9971
Honor	0. 8351	1.0000	0.9245	0.9282
Xiaomi	0.9713	1.0000	0.9963	0.9968

**Table 6:** Accuracy comparison of models with different learning rates.

Phones	0.01	0.1	1	0.001	0.0001
iPhone	0.9571	0.4843	0.4686	0.9119	0.7107
vivo	1	0.7578	0.5214	0.9972	0.9886
Honor	0.9602	0.7706	0.4434	0.9541	0.9541
Xiaomi	0.9966	0.7148	0.4880	0.9691	0.9794

**Table 7:** Accuracy comparison of models with different optimizers.

Phones	${Adam}_{acc}$	${SGD}_{acc}$	${ASGD}_{acc}$	${Adagrad}_{acc}$
iPhone	0.9528	0.5844	0.6132	0.8585
vivo	1	0.9744	0.9487	0.9915
Honor	0.9908	0.8899	0.9450	0.9450
Xiaomi	1	0.9381	0.9588	0.9794

**Table 8:** Running time comparison of models with different experimental environments.

Experiment Environment	CPU	${\mathrm{Model}\;\mathrm{Training}}_{\mathrm{time}}$(s)	${\mathrm{Model}\;\mathrm{Test}}_{\mathrm{time}}$
Experiment environment 1	AMD Ryzen 5 4600U	125	20
Experiment environment 2	AMD Ryzen 7 3750H	194	23

## Data Availability

The original contributions presented in the study are included in the article, further inquiries can be directed to the corresponding authors.
